# Patient perspectives on multidimensional learning and person-centred care: interviews with persons living with type 2 diabetes

**DOI:** 10.1080/02813432.2024.2423881

**Published:** 2024-11-04

**Authors:** Alma Dautovic, Eva Brink, Susanne Andersson, Ulla Fredriksson-Larsson

**Affiliations:** a Department of Health Sciences, University West, Trollhättan, Sweden; b FoU-Centrum Skaraborg – R&D Centre Skaraborg, Skövde, Sweden

**Keywords:** Diabetes specialist nurse, diabetes care, eHealth, patient experiences, professional–patient relations, work-integrated learning

## Abstract

**Objective:**

The objective of this study was to explore patients’ learning and support needs within contemporary diabetes care to help them deal with daily life challenges.

**Design:**

A qualitative descriptive design was used following the Consolidated Criteria for Reporting Qualitative Studies. The 15 individual face-to-face interviews were analysed using qualitative content analysis.

**Setting:**

Participants were drawn from three healthcare centres in rural and urban regions of West Sweden.

**Participants:**

The study involved 15 patients (8 men, 7 women) with T2DM who experienced contemporary diabetes care.

**Results:**

Patients expressed a strong desire for access to person-centred, multidimensional learning, with a focus on genuine partnership, tailored education, and emotional engagement. Digital tools were seen as valuable aids in their self-care efforts. Sub-themes were ‘*Desiring genuine partnership and tailored patient education*’ and ‘*Needing support related to altered perspectives on life and awareness of care standards but with finite care resources*’.

**Conclusion:**

The findings suggest that integrating person-centred, multidimensional learning strategies into diabetes care could be beneficial, particularly when addressing both practical and emotional needs. Encouraging active patient engagement through flexible digital solutions and providing support for emotional well-being may improve the overall patient experience. However, further research and practical application are needed to fully understand how these strategies could be effectively implemented to support patients with T2DM in managing their daily health challenges.

## KEY POINTS

- The participants desired multidimensional learning and person-centred care according to their capabilities and needs.
- The participants wanted to be acknowledged as capable persons who contributed to care when encouraged by professionals.
- Cross-checking and comparing information from multiple sources are common approaches to consider in future diabetes care.

## Introduction

The global prevalence of type 2 diabetes mellitus (T2DM) is steadily increasing, including in Sweden, and the condition remains a significant healthcare concern [1]. Person-centred care has surfaced as a promising means to manage patients’ diabetes and provide high-quality care without increased costs. Healthcare based on this approach considers patients’ goals and embraces their capabilities and available healthcare resources [2].

The Gothenburg Framework for Person-Centred Care consists of three routines that have been widely implemented in daily healthcare practice to facilitate the initiation, integration, and safeguarding of person-centred care [2–4]. The first routine involves developing partnerships with patients by listening to their narrative accounts of the illness, its symptoms, and how it affects their lives. The second routine involves working collaboratively to effect shared decision-making and set mutually agreed treatment goals. Person-centred care departs from traditional medically oriented goals for diabetes care towards integrating patients’ individual goals into care planning. The third routine safeguards partnerships between patients and professionals by documenting patients’ narratives, resources, and agreed-upon goals in shared care plans [4]. A systematic review of randomised controlled trials suggested that allowing patients and healthcare providers to share information can significantly improve patient outcomes [5]. Such exchanges should be tailored to improve patients’ comprehension of their conditions and encourage self-care. This can be achieved by providing personalised treatment plans to encourage patients to take responsibility for medication adherence and lifestyle decisions. A review revealed that there has been a shift in diabetes treatment towards person-centred care [2]. This approach emphasises the need for accessible, relevant, and disease-specific educational materials; effective communication; and enhanced provider resources. It is essential to prioritise patients’ needs and resources in care plans rather than relying on standard care provision. Moreover, care plans should be developed and shared goals established to ensure that care provision meets individuals’ needs and resources [4]. In a qualitative study, patients with type T2DM highlighted the need for flexible ways to receive information and education about their condition [6]. They emphasised that everyone is unique and learns differently, so personalised learning opportunities that integrate current knowledge, new research, and technologies are essential. This study [6] also showed that continuity of care is crucial for allowing patients to continue learning about their condition from the healthcare providers they trust.

In Sweden, the National Board of Health and Welfare provides national guidelines for diabetes care that are intended to provide high-quality equal access to care [7]. Most patients with T2DM visit their healthcare physicians and diabetes specialist nurses annually for diabetes management [7,8]. Diabetes complications can reduce quality of life, increase disability, cause premature death, and exacerbate healthcare costs. Diabetes treatment should focus on enhancing patients’ independence and self-care, which require personal knowledge, skills, and commitment to prevent complications and maintain quality of life [9].

Within self-care diabetes frameworks, diabetes specialist nurse crucially assist persons in harnessing their abilities and overcoming barriers. Patients may need help identifying the requirements for a healthy lifestyle, including diet choices, exercise, glucose monitoring, medication, and problem-solving skills, which are beneficial for self-care. Continuous learning may be crucial for improving health and supporting individuals in developing problem-solving skills, adopting healthier behaviours, and adjusting their care plans based on new knowledge [10].

Learning is a holistic individual human process involving three co-occurring (content-, incentive-, and interaction-based) processes. The content dimension comprises knowledge and skills that enable learners to construct meaning and develop the ability to deal with practical life challenges. The incentive dimension includes the feelings, volitions, and motivations necessary for learning to take place. These two processes are always initiated by impulses in the interaction dimension, which can manifest as perceptions, experiences, activities, or participation in social situations [11,12].

Sweden aims to digitalise and modernise healthcare through eHealth to provide high-quality care for all citizens [13]. eHealth refers to ‘the use of information and communications technology in support of health and health-related fields’ [14], including diabetes. Patients with T2DM already benefit from eHealth systems (e.g. mobile phone apps and virtual consultations via email, telephone, or webcam), which have been shown to improve treatment outcomes [15]. Prior research has also revealed that patients with T2DM need personalised support to manage their condition effectively and lead satisfying lives despite the disease [16]. Personalised communication to strengthen patients’ engagement in self-care is a vital component of diabetes care. Significantly, digital technology contributes to the continual development of digital tools and the ready availability of health information to enhance the quality of life of persons living with T2DM. More research is needed regarding ways to ensure that patients are sufficiently informed and involved in issues related to living with T2DM. Therefore, this study aimed to explore patients’ learning and support needs within contemporary diabetes care to help them deal with daily life challenges.

## Materials and methods

This study used a qualitative descriptive approach. Specifically, 15 individual qualitative interviews were conducted and systematically analysed using qualitative content analysis [17–19]. The qualitative design was evaluated using the Consolidated Criteria for Reporting Qualitative Studies checklist [20] (see Supplemental Appendix 1).

### Setting and participants

The study context comprised three healthcare centres in rural and urban areas of West Sweden, selected through collaboration between the university and the region. An invitation to participate was sent to all healthcare centres in the area. Initially, four healthcare centres expressed interest in participating, but one withdrew due to the COVID-19 pandemic workload.

Five diabetes specialist nurses recruited the participants for the study when they contacted or visited the participating healthcare centres. The convenience sample comprised 15 participants, primarily Swedish (8 men and 7 women), with a mean age of 68 years; see Table 1. The inclusion criteria were as follows: (1) diagnosed with T2DM, (2) English- or Swedish-speaking, and (3) registered at one of three healthcare centres in West Sweden. Patients unable to speak due to language difficulties or cognitive impairments were excluded.

**Table 1.** Characteristics of the participants.

Participants	Age	Gender	Diabetes duration	Medical treatment	Estimated digital use*: minutes/day	Continuous glucose monitor	Urban/Rural area**	Origin
1.	53 years	Male	15 years	Tablets	180		Urban	Non-Swedish
2.	79 years	Male	10 years	Tablets and insulin injection	60		Urban	Swedish
3.	60 years	Female	10 years	Tablets and multiple insulin injections	720		Rural	Swedish
4.	67 years	Female	1 year	Tablets	60		Urban	Swedish
5.	71 years	Male	20 years	Tablets, multiple insulin injections, and GLP-1 analogue injection	120		Urban	Swedish
6.	72 years	Female	11 years	Tablets and insulin injection	90		Urban	Swedish
7.	75 years	Male	10 years	Tablets	30	x	Rural	Swedish
8.	79 years	Male	22 years	Tablets, multiple insulin injections, and GLP-1 analogue injections	30		Rural	Swedish
9.	69 years	Male	5 years	Tablets	120		Urban	Swedish
10.	70 years	Female	22 years	Tablets and GLP-1 analogue injection	30		Urban	Swedish
11.	74 years	Female	9 years	Tablets	30		Urban	Swedish
12.	67 years	Male	18 years	Tablets	240		Rural	Swedish
13.	28 years	Female	4 years	Tablets, multiple insulin injections, and GLP-1 analogue injections	350	x	Rural	Swedish
14.	70 years	Male	1,5 years	Tablets	180		Urban	Swedish
15.	82 years	Female	25 years	Tablets, insulin injections, and GLP-1 analogue injections	10		Urban	Swedish

* Computer, reading tablet, or smartphone. **A municipality with at least one large city with a population of 30,000 is considered an urban area. In contrast, a municipality with a population of less than 15,000 is considered a rural area [38].

### Data collection

The data for this study were collected via semi-structured interviews. The research team developed a semi-structured interview guide with open-ended questions to ensure that all topics – diabetes, diabetes care, the role of diabetes specialist nurses, learning, digitalisation, and general health – were discussed in all the interviews. The exact wording of the questions was not predefined, and additional follow-up questions were used to clarify uncertainties, obtain further details, and widen the interview scope. Probing questions, such as ‘Could you tell me more about that?’ [21], were asked when appropriate to enhance understanding of the participant’s replies. The interviews lasted 40–75 min (mean 63.5) and were audio recorded. They were conducted in Swedish by the first author (AD) at the patients’ respective healthcare centres between May and July 2022. Regular conversations took place between AD and a senior experienced researcher (EB), and interviews were discussed throughout the interview period to ensure that the interviews were of high quality and to interpret the compiled data. When the participants began to repeat recognisable information in their statements, the authors made a joint decision to cease the data collection, which left 15 evenly distributed participants (i.e. five from each participating healthcare centre). An increased sample size or number of data text pages does not necessarily enhance data richness [22].

### Ethical considerations

The Swedish Ethical Review Authority approved this study (Dnr. 2022-00790-01), and the researchers followed the Helsinki Declaration of Medical Research Ethics and Standards [23]. The contact with participants was established through five diabetes specialist nurses who worked at the three participating healthcare centres and invited the patients to participate. The diabetes specialist nurses’ gave potential participants written and oral information about the study. Participants were contacted and involved in the study only after they signed informed consent forms.

After obtaining the participants’ signed informed consent by mail, the first author (AD) contacted them by telephone, addressed potential questions, and scheduled individual face-to-face interviews.

### Data analysis

The data analysis began when all 15 interviews had been conducted and transcribed verbatim by the first author (AD). The text was subjected to qualitative content analysis via a structured nonlinear process in which the researchers moved back and forth between the original text and various extracts from the text [17–19]. Each interview transcript was read thoroughly several times by the first author (AD), with and without audio, to facilitate immersion in the data. The NVivo software program [24] was used during the entire data analysis process. All co-authors had access, and each process step was followed thoroughly. NVivo facilitates nonlinear text analysis by allowing users to switch easily between excerpts and the whole text within the programme, ensuring a transparent analysis for the co-authors.

During the analysis, the transcripts were marked line by line to identify meaning units that corresponded to the study aim, condensed (i.e. shortened while still preserving their core content), and coded with descriptive labels that reflected the original text [17–19]. This was done by the first author (AD) in close collaboration with the co-authors (EB, SA, and UFL). The research team’s diverse blend of academic and nursing experiences (Supplemental Appendix 1) enhanced and added depth to the analysis since all co-authors participated in the entire analysis, which minimised bias [25].

Qualitative content analysis focuses on subjects and contexts in data and emphasises differences and similarities within codes and categories [17]. In this case, the diverse codes were abstracted, interpreted, and assigned to descriptive content categories using a decontextualising process [18]. Another characteristic of content analysis is that it considers both manifest and latent textual content: categories that contain manifest descriptive content arise from the data with varying degrees of abstraction and low degrees of interpretation, while themes are seen as expressions of the latent, underlying content that crosses several categories and thus requires deeper interpretation [17–19]. Despite initial disagreement regarding some interpretations, the authors achieved consensus after reflection and discussion. Researchers’ interpretative repertoires can vary, so the consensus is defined as co-creative analysis by more than one researcher [26]. Interpretation is part of the recontextualising process, moving from descriptions of the manifest content to the latent content and the creation of different interpretive themes (Table 2). Interpretation of the data is a critical step in the content analysis process, and collaborative analysis and interpretation are recommended to prevent over-interpretation [18].

**Table 2.** Description of the data analysis procedure, presenting the main team, sub-themes, and categories.

Main theme	Sub-themes	Categories
A desire to have access to multidimensional learning and person-centred diabetes care	*Desiring genuine partnership and tailored patient education*	*Expressing the need for flexible learning strategies*
		*Using available digital tools to promote personal learning activities and interactions*
		*Asking for authentic listening and genuine professional dialogue*
		*Appreciating familiarity and continuity-driven diabetes care*
	*Needing support related to altered perspectives on life and awareness of care standards but with finite care resources*	*Changing viewpoints on how to lead one’s daily life*
		*Desiring that the received care aligns with the national guidelines for diabetes care*
		*Experiencing limited diabetes care support*

## Results

The analysis resulted in the main theme of *‘A desire to have access to multidimensional learning and person-centred diabetes care’*. Our study identified that multidimensional learning was closely tied to person-centred care, where both approaches addressed the complex needs of each patient as a person. This learning approach emphasises the importance of creating learning environments that are not only intellectually stimulating, but also emotionally supportive and practically relevant to persons living with diabetes. This learning approach advocates for a genuine partnership between healthcare professionals and patients, wherein tailored patient education is able to meet the patients’ individual needs. The sub-themes identified in our analysis – *‘Desiring genuine partnership and tailored patient education’* and *‘Needing support related to altered perspectives on life and awareness of care standards, but with finite care resources’* – are integral to this concept. They highlight the patient’s desire for a more engaged and personalised educational experience that encourages reflection on personal values and life goals while recognising the practical limitations imposed by the available resources. Ultimately, multidimensional learning can enhance the efficacy of person-centred care when patients are recognised as capable and equal contributors to their care and learning.

### Desiring genuine partnership and tailored patient education

#### Expressing the need for flexible learning strategies

The participants expressed their desire for flexible learning, which meant accessing information from various sources at different times to acquire knowledge according to their needs. They found small amounts of information educative, particularly if repeated on subsequent occasions. They found that professionals who encouraged them to reflect on their experiences or questions helped them develop solutions that aligned with their unique condition. Reflection on daily situations was crucial for personal growth and learning to manage diabetes.

Receiving important health-related information verbally from diabetes specialist nurses seemed to be the preferred source of learning. Verifying and cross-checking information by comparing content across information sources was considered an essential step in the flexible learning process:

Of course, I also looked it up [information given by a diabetes specialist nurse] on the internet afterwards and read about it. I found it was correct. (Participant 14)

A standard cross-checking method was discussing the obtained information with people who had experienced comparable situations in person or via online forums. Some participants conducted independent research on diabetes-related topics before meeting with professionals and cross-checked the information with them during the meetings.

Despite participants recognising the benefits of involving family members in their flexible learning strategies to promote learning and self-care, professionals rarely invited them to appointments. Participants felt despair when family members lacked awareness of the disease, emphasising the need for greater family involvement in patient education because they relied on family interventions at home when necessary:

If relatives don’t know anything about it [the disease], you’re doomed. … Where can you get help if you need it? She [his wife] can see when I become dizzy … because of an infection. (Participant 12)

#### Using available digital tools to promote personal learning activities and interactions

Patients appreciated the advantages of digital technology, particularly sensors for continuous blood glucose monitoring. Although only two participants had access to such tools (Table 2), many more expressed interest in them and recognised the potential benefits of using them to improve their self-care and educational outcomes. One participant who required multiple insulin injections but did not have access to continuous blood glucose monitoring was especially keen to acquire a digital tool that could simplify life and enhance learning:

I would see that as an advantage, to have an app [on the smartphone] and just be able to check [the glucose level]. And something that would be good, with just a button [continuous glucose meter] and the app, is that you could follow up on [glucose] after meals. Did I take 12E [units of insulin] for this dinner I ate? Was it enough? Then, I would know that the next time I ate roughly the same thing, the dose wasn’t enough. That would absolutely make life easier. It would simplify life in general and help me maintain a more even blood sugar level. (Participant 3)

Participants expressed the need to increase the availability of digital diabetes tools (e.g. sensors and apps) for T2DM patients and to enable them to obtain reliable online diabetes-related information. They claimed that the quality of online diabetes information varied and could be substantially improved. The participants generally sought guidance from professionals on trustworthy websites, and online information could overcome language barriers by incorporating translation features:

If a website, for example, is in Arabic, English, or Swedish, you can live here [in Sweden] and get information in English if you understand English better. (Participant 1)

Some participants stated that the increased availability of online information and different ways of interacting in online forums gave them new ideas about treatment strategies and changes. The participants expected professionals to listen, consider suggestions, and potentially learn from patients, but not all professionals appreciated patients’ influence on healthcare:

The first time I came here, I met a diabetes specialist nurse; she was close to retirement age, and it was hard to talk to her … She was the kind of person who looked down on you. It is often like that in healthcare, I feel. … If you make suggestions, [they reply], ‘No, I’m trained in this, I know this’ … but it hasn’t been like that with my current nurse. (Participant 13)

Patients appreciated the opportunity to use the National Healthcare Guide’s online platform to access their electronic patient records, schedule appointments, renew prescriptions, and communicate with healthcare professionals instead of using the traditional call–recall telephone system. The participants expressed a desire for more direct digital communication with healthcare professionals:

[I wish] I could e-mail a question, like you do in all other places, and … get a quick answer. Almost like a chat … where you get the reply immediately. That’s when you might need the response quickly—when you have a problem. (Participant 9)

#### Asking for authentic listening and genuine professional dialogue

The participants saw diabetes specialist nurses as crucial allies in their quest for autonomy. They desired helpful, rational, sensible, involved, friendly, curious, and dedicated professional partners. They preferred approaching a friendly diabetes specialist nurse who considered their unique needs and offered personalised counselling over impersonal responses. They mentioned that if they were personally convinced by the advice they received, they would probably adopt it and work towards enhancing their daily lives. The participants preferred to interact with enthusiastic and interested diabetes specialist nurses, whom they perceived as being more committed to offering above-and-beyond care:

There should be interest—not just that you should do it [routine work], take tests, and say goodbye. That has happened. (Participant 12)

I know it’s not easy to change old men, but she can try, and it’s probably a tiresome job. Furthermore, it might be tiresome for her, but it is necessary. (Participant 5)

The participants held diabetes specialist nurses in high esteem when they used their knowledge and professional skills to improve health outcomes through professional dialogue:

She is responsive, I must say. I think that’s an advantage she has—that she knows a lot and is responsive at the same time. (Participant 2)

#### Appreciating familiarity and continuity-driven diabetes care

Participants found that having consistent staff at their healthcare centres made it easier to build trust with professionals. Participants who interacted with the same staff members felt valued, seen, and recognised as a part of the diabetes team:

If you go to the same person, they see you differently. (Participant 6)

I am not just a name. I AM MARGRETE! [pseudonymised] (Participant 10)

The feeling of continuity and familiarity gave the participants confidence that professionals would be able to identify changes in their health sooner and assist them in avoiding unfavourable consequences:

They can see that you feel a bit worse, and they can also see that you feel a bit better without feeling really good or bad. They can recognise shifts that no one else can and that you don’t notice the first time you meet. (Participant 6)

Patients who experienced a sense of familiarity and togetherness in their healthcare centres tended to use the pronoun ‘we’ to refer to the professionals with whom they interacted. This indicated shared responsibility and collaboration in their quest for improved health. However, participants who were unfamiliar with professionals tended to refer to them using the pronoun ‘they’. This implied a lack of shared care responsibility and indicated that these participants handed over the disease liability to the staff:

I’ve been doing that for a long time, lying about it [a blood sugar level of 10]. So that’s what they are considering because it had gone up last time … At the long-term sugar check … the nurse checks with the doctor whether they should increase the [medication]. (Participant 7)

Patients reported receiving fragmentary advice and contradictory recommendations when seeking healthcare; typically, professionals’ advice was based solely on the patients’ symptoms and was not integrated into their overall healthcare:

For diabetes, the advice is that I should exercise, but … for my knees and hips, [the physiotherapist] says, ‘You shouldn’t walk so much and should rest’. There’s not much cooperation between them … They may only read about how their knowledge applies to my case … and going into the other areas may not be comfortable for them. (Participant 4)

The participants preferred healthcare professionals who understood the impact of different health problems and could provide person-centred care.

### Needing support related to altered perspectives on life and awareness of care standards, but with finite care resources

#### Changing viewpoints on how to lead one’s daily life

A diabetes diagnosis can alter a person’s perspective on life, particularly when the individual compares his or her life before and after diagnosis. Lifestyle modifications and needs vary; some people require minor adjustments, while others need significant life-altering changes that involve new insights. By gaining new insights, patients can make informed and healthy decisions based on their knowledge and earlier experiences, enabling them to navigate similar situations more confidently:

The brain is a bizarre contraption because you can eventually get used to the fact that you’re not tempted by your husband going to the ice cream stand. (Participant 10)

The participants sought safe and supportive conversations with healthcare professionals and opportunities to share their feelings and experiences and to develop strategies for tackling social stigma. Preconceived assumptions that T2DM is prevalent only in older, overweight individuals can make patients feel insecure. Sometimes, these feelings of uncertainty were triggered by comments from professionals:

The doctor told me that many people get diabetes because they are overweight and said, ‘I do not understand how someone like you, [patient name], could have got diabetes when you are not that fat’. (Participant 8)

The participants described feelings of embarrassment, guilt, humiliation, failure, being different, and feeling left out when surrounded by people who did not understand the adaptations necessary for maintaining daily life:

If you are invited out, you must think about it. … It’s the case nowadays that people who do not have [diabetes] do not have unsweetened cookies and expect you to eat everything that is set out, and if you do not take anything, you feel left out. (Participant 15)

To change their viewpoints and reduce stigma, the participants needed supportive learning environments in which they felt comfortable sharing their experiences of illness, leading them to new insights.

#### Desiring that the received care aligns with the national guidelines for diabetes care

To ensure nationally equitable diabetes care, the participants were aware of the national guidelines for diabetes care and acknowledged that they should be followed. They explained that the guidelines were followed in terms of frequent follow-ups and coordination with diabetes specialist nurses in cases of elevated glycosylated haemoglobin. Diabetes specialist nurses then provided critical support for reducing blood sugar levels, which patients highly appreciated. Nevertheless, they said that the national guidelines were not adhered to during diagnostic procedures, which led to delayed diagnoses for some participants. They lamented the time it took to receive a diagnosis, believing that some disease-related consequences might have been avoided:

I was never told by a doctor that I had diabetes, not until he [the ophthalmologist] said that I probably had diabetes. And that was two years after this [uncertain diagnosis]. (Participant 11)

Due to shortages of diabetes specialist nurses, some patients have not visited diabetes specialist nurses for several years, despite the fact that the national guidelines for diabetes care insist on regular visits. These patients were given the option of annual checkups with physicians instead, but they preferred to visit both types of specialists annually. The patients noticed inequality in the distribution of qualified diabetes specialist nurses and objected to this disparity in resources nationwide. The participants lauded digitalisation as a viable strategy for reducing geographic inequalities and ensuring adherence to national guidelines for diabetes care. However, both patients and professionals in geographical areas that necessitate long travel distances to healthcare centres may need extended digital competencies:

If I could not … travel 40 miles [referring to the Swedish mile, 400 kilometres] to visit the health centre or doctor, these skills would be essential. I think you should have the opportunity to ‘visit’ via telephone, computer, or tablet. (Participant 5)

#### Experiencing limited diabetes care support

The growing number of people with diabetes has left many patients feeling concerned about their limited access to quality diabetes care. Some participants noted that the lack of diabetes care in healthcare centres is mainly due to the unavailability of diabetes specialist nurses. Attracting nurses with specific diabetes knowledge to healthcare centres was perceived as a challenge. When participants with high glucose levels faced limited diabetes care, they used two strategies to overcome it: the first was to call the national healthcare guidance number, which schedules urgent appointments at the patient’s healthcare centre; the second was to inform the healthcare centre about other medical conditions that required immediate attention:

I checked my blood pressure [at home], and there was an irregular pulse, so I called and got an appointment with the doctor … I had a blood sugar level of 20 … and he [the doctor] prescribed insulin. I had to start using insulin. (Participant 11)

Not all participants developed strategies to deal with limited care support. Instead, some waited for appointments, even after informing their healthcare centres about problems with elevated blood sugar levels:

I pricked my finger … and the monitor showed HIGH; I could not even see the blood sugar level, but it took two months before I got an appointment, and that was when I was sent [to a local hospital]. (Participant 13)

## Discussion

The study revealed that the participants had diverse multidimensional learning needs. Multidimensional learning involves using different methods to comprehend and assimilate vital health information. This approach is similar to a holistic learning perspective encompassing content, incentives, and interaction-based processes [11]. This study showed that diabetes specialist nurses can help patients improve their diabetes self-care confidence through multidimensional learning. Cognitive learning involves encouraging patients to reflect on provided information, whether acquired independently online or provided orally by healthcare professionals, to help them develop solutions to everyday problems. The participants expressed a desire for healthcare providers to recognise the knowledge they gained from their own learning activities. They appreciated professionals who considered them capable active partners who could formulate their own solutions to health problems. Professionals should view patients as persons and support their desire to feel like team members [27]. Professionals should engage with patients and confirm their capabilities to establish patient partnerships. From a person-centred perspective, even though patients may suffer, as in this case, from a long-term disease, the suffering should never be considered debilitating. Instead, humans should be seen as capable of developing actions to face suffering [28]. Inclusivity is crucial in diabetes care because it creates a sense of togetherness and encourages patients to take ownership of their illness. Patients experienced a shared diabetes responsibility when they felt included, and they claimed that connecting with healthcare professionals was essential for promoting good health. To achieve this, patients’ capabilities and needs should be identified. Embracing a person-centred approach in diabetes care allows patients to learn and grow on their own terms through multidimensional learning practices.

The incentive and interaction processes [11] in the current study were related to the emotional and social dimensions of learning concerning the feelings and social difficulties that patients experience when altering their perspectives on life after a diabetes diagnosis. The patients explained that contemporary diabetes care does not commonly have these two dimensions of multidimensional learning. In this study, the participants stated that the disease’s emotional aspects and stigma were significant concerns for them. They preferred to discuss their feelings, as they believed this was crucial for promoting their learning processes. They asserted that patient education should place more emphasis on emotional support. The participants felt alone in dealing with their feelings and did not know to whom they should turn. They needed professionals who could help them learn how to regulate the adverse emotions they experienced and encourage them to build healthy social relationships despite illness. One study reported that patients with diabetes often struggle to follow recommended medical treatments due to social and emotional challenges that distract them from self-care [29]. To successfully promote learning and self-care among people with diabetes, professionals should recognise that feelings and social experiences greatly influence patients’ learning processes.

In contemporary diabetes care, patients desire person-centred multidimensional learning but may encounter difficulties if healthcare organisations follow rigid standardised approaches that fail to acknowledge patients as unique persons. Some participants reported that healthcare organisations still use checklist-based routines, which can hinder the implementation of person-centred care and multidimensional learning. Such implementation calls for a perspective change regarding how diabetes care is provided and requires professional retraining and the adoption of new strategies for interacting with motivated, capable persons. Diabetes specialist nurses can motivate action-oriented patients through genuine dialogue and effective listening. According to a previous qualitative study [3], professionals’ clinical mindsets need to shift towards recognising patients as capable, engaged team members regarding their treatment. Identifying a person as resourceful and capable despite their disease and promoting healthcare based on their own experiences is a professional responsibility that requires flexible and open-minded treatment approaches. In line with our findings, another qualitative study demonstrated that patients frequently seek online health information to support them in understanding difficult healthcare decisions [30]. Accordingly, professionals should show interest in patients’ online practices and acknowledge the potential health benefits of online health information that may otherwise be lost.

The present study contributes content from the patients’ perspectives. The category ‘*Desiring that the received care aligns with the national guidelines for diabetes care’* reflects the patients’ expectations for quality care, which they associated with adherence to national standards [7]. While it may not be expected for all patients to have an in-depth understanding of the national guidelines, there is at least a general expectation that the care they receive is up-to-date, evidence-based, and reflective of the best practices described in the guidelines. However, several participants asserted that they were ineligible for annual diabetes specialist nurse visits because their healthcare centres did not have diabetes specialist nurses. The participants claimed a deficiency in this respect and proposed that the increased use of digital tools would ensure more equitable diabetes care. They were familiar with the Swedish online national platform, which provides information and services. They used it frequently to review their patient records, obtain up-to-date information, and interact digitally with professionals. However, they suggested introducing a nationally regulated diabetes-focused website and more direct physical or digital communication with diabetes specialist nurses. A literature review [31] has promoted eHealth as a potential resource in healthcare systems to encourage self-care. Patients have been found to perceive communication with professionals via electronic-based systems as flexible and time-saving [31,32]. Generally, eHealth systems have been recognised as helpful for diabetes treatment by both patients and professionals, and diabetes specialist nurses should integrate eHealth into diabetes treatment in future diabetes care [31].

The participants claimed that family involvement was also important, and they found it easier to follow recommendations with family support, although professionals hardly ever invited family members to engage in their diabetes care. A review study [33] highlighted that social support provided by family members is essential for ensuring effective diabetes care and can be critical for overcoming barriers and sustaining self-care. With good family support, patients are better equipped to manage their everyday lives and can significantly improve their clinical outcomes (e.g. HbA1c, blood pressure, and BMI); therefore, professionals should strive to include family members to ensure a higher level of diabetes care.

Finally, the results showed that the participants felt competent and powerful when using digital technology to obtain up-to-date information. Collaborating with action-oriented patients entails adopting new working practices and work-integrated learning possibilities and recognising patients as equally capable people. In a focus group interview study [34], the healthcare professionals reported that patients’ group education increased their own knowledge because they learned from patients’ experiences. Professional development is shaped by work-integrated learning that arises from work situations and reflections on experiences [35].

## Strengths and weaknesses of the study

A strength of this study lies in its methodology. The findings’ credibility, confirmability, dependability, and transferability to ensure the study’s trustworthiness [17,18,36] are discussed in the following paragraphs.

Another of the study’s strengths is its diverse participant sample, including various genders, ages, medication strategies, diabetes duration experiences, and geographical areas. This variation allowed the authors to examine multiple perspectives on contemporary diabetes care, confirming the study’s credibility. The interviews were based on predetermined topics and open-ended questions. The interviewer clarified any misunderstandings by asking supplementary questions. A possible limitation was that the interviews were conducted and transcribed in Swedish, although English translations were conducted after the meaning units had been categorised. This translation may have affected some data. However, the translation was performed by an authorised English translator.

Regarding confirmability, there was a possibility of social desirability bias in this study. The participants did not know the interviewer before the study, but when requested, the interviewer introduced herself as a researcher and diabetes specialist nurse. Additionally, the interview questions and data collection were based on clinical empirical work, which could have impacted the research questions and the interpretation of the data. To mitigate this potential limitation, the interviews were coded and analysed using NVivo, and the analysis was conducted by four researchers with different nursing and academic experiences. The research thus benefited from being conducted by multiple researchers, which created a wider analytical framework [37] and ensured the dependability of the findings [26]. All co-authors were able to follow the analytical process in NVivo and reach a common understanding of the coding strategy. Finally, all the authors discussed and iteratively revised the categories. It is crucial to bear in mind that some interpretation is always involved when using qualitative analysis and no descriptions are free from interpretation [19]. Therefore, to ensure the trustworthiness of the research findings, researchers need to consider ways to achieve credibility and confirmability. This paper includes direct participant quotations to illustrate the results and support the findings’ confirmability.

This study was conducted during the COVID-19 pandemic when Sweden’s healthcare system faced great pressure that made it difficult to recruit participants of non-Swedish origin. Despite our best efforts, we could only recruit one non-Swedish-origin participant, which we acknowledge as a limitation of this study.

## Implications

Policymakers and practitioners in nations moving towards digitalised person-centred care may find the results of this study helpful. The findings indicate that people with diabetes are action-oriented and want to play significant roles in their care if professionals give them the opportunity. However, the care arena needs to be reshaped to maximise this possibility. A potential way to address this would be for professionals to provide multidimensional learning opportunities to match patients’ needs and capabilities and acknowledge them as capable humans. Persons with T2DM perceive eHealth use as a natural component of self-care, which may enhance partnerships with healthcare professionals. By using digital technology to exchange updated diabetes care information, patients and professionals may gain new insights that promote person-centred care and a sense of togetherness.

## Supplementary Material

Supplemental Material

## Data Availability

No unpublished data are available following this study.
