# Supplementary material for: Patient perspectives on multidimensional learning and person-centred care: interviews with persons living with type 2 diabetes
Source: Scand J Prim Health Care. 2024 Nov 4;43(1):230–40. doi: 10.1080/02813432.2024.2423881 (PMC11834784; doi:10.1080/02813432.2024.2423881)
Supplement: Supplemental Material [file IPRI_A_2423881_SM9755.docx]

**Consolidated criteria for reporting qualitative studies (COREQ): a 32-item checklist.**

| **No. Item** | **Guide questions /description** |  | **Reported on Page #** |
| --- | --- | --- | --- |
| **Domain 1: Research team and reﬂexivity** |  |  |  |
| ***Personal Characteristics*** |  |  |  |
| 1. Interviewer/facilitator | Which author/s conducted the interview or focus group? | The first author (AD) conducted the interviews. | 8. |
| 2. Credentials | What were the researcher’s credentials? E.g. PhD, MD | The researchers’ credentials are as follows:  AD: PhD student.  EB: PhD  SA: PhD  UFL: PhD | N/A. |
| 3. Occupation | What was their occupation at the time of the study? | The researchers’ occupations are as follows:  AD: RN, PhD student,  EB: RN, PhD, senior professor.  SA: RN, PhD, associate professor  UFL: RN, MSc, senior lector.  All have previous extensive nursing experience for many years. | 10. |
| 4. Gender | Was the researcher male or female? | The researchers’ gender are as follows:  AD: female  EB: female  SA: female  UFL: female | N/A. |
| 5. Experience and training | What experience or training did the researcher have? | EB, SA and UFL are experienced researchers in qualitative studies and have collectively published numerous qualitative research articles.  AD attended methodological courses on “how to conduct qualitative research” and “how to use NViVo software to analyse the data”. | N/A. |
| ***Relationship with participants*** |  |  |  |
| 6. Relationship established | Was a relationship established prior to study commencement? | Only for the purposes of this research. | 9. |
| 7. Participant knowledge of the interviewer | What did the participants know about the researcher? e.g. personal goals, reasons for doing the research | Although the participants did not know any of the researchers, they all knew that the interview was for research purposes. | 23. |
| 8. Interviewer characteristics | What characteristics were reported about the interviewer/facilitator? e.g. Bias, assumptions, reasons and interests in the research topic |  | 8,10, 23. |
| **Domain 2: study design** |  |  |  |
| ***Theoretical framework*** |  |  |  |
| 9. Methodological orientation and Theory | What methodological orientation was stated to underpin the study? e.g. grounded theory, discourse analysis, ethnography, phenomenology, content analysis | A qualitative descriptive study with qualitative content analysis. | 7. |
| ***Participant selection*** |  |  |  |
| 10. Sampling | How were participants selected? e.g. purposive, convenience, consecutive, snowball | Participants were collected through a convenience sample; specialist diabetes nurses at participating healthcare centres invited participants. | 8. |
| 11. Method of approach | How were participants approached? e.g. face-to-face, telephone, mail, email | Five specialist diabetes nurses at participating healthcare centres provided potential participants with oral and written information about the study. First, after sending written consent and contact information to the research team, the first author, AD, contacted informants to schedule an interview. | 8-9. |
| 12. Sample size | How many participants were in the study? | Fifteen informants from three different healthcare centres were included in the study. | 8-9. |
| 13. Non-participation | How many people refused to participate or dropped out? Reasons? | None of the informants asked to participate dropped out. | N/A |
| ***Setting*** |  |  |  |
| 14. Setting of data collection | Where was the data collected? e.g. home, clinic, workplace | Data was collected at patients’ healthcare centres. | 8. |
| 15. Presence of non-participants | Was anyone else present besides the participants and researchers? | No one else was present besides the participants and the researcher AD. | N/A. |
| 16. Description of sample | What are the important characteristics of the sample? e.g. demographic data, date | The inclusion criteria were 1) diagnosed with T2DM, 2) English- or Swedish-speaking, and 3) registered at one of three healthcare centres in West Sweden. Patients unable to speak due to language difficulties or cognitive impairments were excluded. | 8. |
| ***Data collection*** |  |  |  |
| 17. Interview guide | Were questions, prompts, guides provided by the authors? Was it pilot-tested? | The research team developed a semi-structured interview guide with open-ended questions to ensure that all topics—diabetes, diabetes care, the role of specialist diabetes nurses, learning, digitalisation, and general health—were discussed in all the interviews. The exact wording of the questions was not predefined, and additional follow-up questions were used to clarify uncertainties, obtain further details, and widen the interview scope. The pilot was not tested. | 8. |
| 18. Repeat interviews | Were repeat interviews carried out? If yes, how many? | No repeat interviews were carried out. | N/A |
| 19. Audio/visual recording | Did the research use audio or visual recording to collect the data? | All interviews were audio recorded and transcribed verbatim. | 8. |
| 20. Field notes | Were ﬁeld notes made during and/or after the interview or focus group? |  | N/A |
| 21. Duration | What was the duration of the interviews or focus group? | The duration of the in-depth interviews ranged from 40 to 75 (mean 63.5) minutes. | 8. |
| 22. Data saturation | Was data saturation discussed? | The abstraction and interpretation process went on through joint analysis, including reflections and discussions within the research team (AD, EB, SA, UFL), resulting in seven categories, two subthemes and one main theme. | 10,26. |
| 23. Transcripts returned | Were transcripts returned to participants for comment and/or correction? | The transcripts were not returned to participants for comment or correction. | N/A. |
| **Domain 3: analysis and ﬁndings** |  |  |  |
| ***Data analysis*** |  |  |  |
| 24. Number of data coders | How many data coders coded the data? | Coding was performed in the software program NVivo by the first author (AD) in close collaboration with co-authors (EB, SA, UFL). | 9,23. |
| 25. Description of the coding tree | Did authors provide a description of the coding tree? | An explanation of the data analysis procedure presenting the main team, sub-themes, and categories is provided, also shown in Table 2. | 11, 24. |
| 26. Derivation of themes | Were themes identiﬁed in advance or derived from the data? | The themes deviate from the data. The research team mutually identified higher-order headings of the data content through discussion, interpretation, and abstraction of the texts. They resulted in two subthemes and one main theme. | 11,24,26. |
| 27. Software | What software, if applicable, was used to manage the data? | The software program NVIVO version 14 (Alasoft AB, Gothenburg, Sweden) contained data straightforwardly and made the analysis progress transparent within the research team. | 9,24. |
| 28. Participant checking | Did participants provide feedback on the ﬁndings? | No, participants did not provide any feedback on the findings. | N/A |
| ***Reporting*** |  |  |  |
| 29. Quotations presented | Were participant quotations presented to illustrate the themes/ﬁndings? Was each quotation identiﬁed? e.g. participant number. | Participant quotations were presented in the results and identified with a specific participant number. Table 1 shows participant numbers and characteristics. | 11-19, 26. |
| 30. Data and ﬁndings consistent | Was there consistency between the data presented and the ﬁndings? | Yes, there was consistency between the data presented and the findings. | 11-19, 27. |
| 31. Clarity of major themes | Were major themes clearly presented in the ﬁndings? | Yes, major themes were clearly presented in the findings. | 11-19,27. |
| 32. Clarity of minor themes | Is there a description of diverse cases or discussion of minor themes? | Yes, subthemes and categories are presented. | 11-19,27. |

**Developed from: Tong A, Sainsbury P, Craig J. Consolidated criteria for reporting qualitative research (COREQ): a 32-item checklist for interviews and focus groups. *International Journal for Quality in Health Care*. 2007. Volume 19, Number 6: pp. 349 – 357**
